# PARP-1 promotes tumor recurrence after warm ischemic liver graft transplantation via neutrophil recruitment and polarization

**DOI:** 10.18632/oncotarget.21493

**Published:** 2017-10-04

**Authors:** Shuai Wang, Fa-Ji Yang, Xun Wang, Yuan Zhou, Bo Dai, Bing Han, Hu-Cheng Ma, Yi-Tao Ding, Xiao-Lei Shi

**Affiliations:** ^1^ Department of Hepatobiliary Surgery, Nanjing Drum Tower Hospital Clinical College of Nanjing Medical University, Nanjing, China; ^2^ Department of Hepatobiliary Surgery, The Affiliated Drum Tower Hospital of Nanjing University Medical School, Nanjing, China

**Keywords:** liver transplantation, hepatocellular carcinoma, tumor recurrence, PARP-1, neutrophil

## Abstract

Poly (ADP-ribose) polymerase 1 (PARP-1) is a crucial contributor to exacerbate ischemia and reperfusion (IR) injury and cancer process. However, there is little research into whether PARP-1 affects the hepatocellular carcinoma (HCC) recurrence after liver transplantation. In this study, we investigated the influence of PARP-1 on hepatic neutrophil mobilizing and phenotype shifting which may lead to HCC recurrence after liver transplantation. We found that rats received the grafts with warm ischemic injury had higher risk of HCC recurrence, which was markedly prevented by pharmacological inhibition of PARP-1 after liver transplantation. In mouse models, the up-regulation of PARP-1 was closely related to the greater tumor burden and increased hepatic susceptibility to recurrence after IR injury. The reason was that high hepatic PARP-1 led to increased liver CXCL1 levels, which in turn promoted recruitment of neutrophils. Both blocking CXCL1/CXCR2 signaling pathway and depleting neutrophils decreased tumor burden. Moreover, these infiltrating neutrophils were programmed to a proangiogenic phenotype under the influence of PARP-1 *in vivo* after hepatic IR injury. In conclusion, IR-induced PARP-1 up-regulation increased the hepatic recruitment of neutrophils through regulation of CXCL1/CXCR2 signaling and polarized hepatic neutrophils to proangiogenic phenotype, which further promoted HCC recurrence after transplantation.

## INTRODUCTION

Primary liver cancer is the sixth most frequent cancer and the second leading cause of cancer-related death worldwide. An estimated 782,500 new diagnosed cases and 745,500 deaths occurred in 2012 [1]. Hepatocellular carcinoma (HCC) is the most common histologic type that it accounts for approximately 90% of all primary hepatic malignancies [2]. It is reported that China has about 55% liver cancer deaths worldwide due to the high incidence of chronic hepatitis B [3]. Liver transplantation seems to be an effective therapeutic strategy for HCC patients and provides higher long term survival prospects [4]. The main limitation is the shortage of donor liver. Simple living donor liver transplantation is difficult to meet the growing demands for availability of organs. Therefore, the lack of organs has led to an increase in the use of extended criteria grafts, such as donation after cardiac death (DCD) donors. The use of DCD grafts in the United States has increased up to 5% and is now around 50% in United Kingdom in 2011 [5]. In addition, the 5-year survival rates of DCD liver transplantation is similar to that of conventional donor liver transplantation (65-80%)[5, 6]. However, it is reported that DCD allograft tended to increase the rates of recurrence for HCC candidates [7] and Oldani G, et al has proved it in rodent DCD models for HCC recurrence after liver transplantation [8].

DCD livers are prolonged exposure to warm ischemia and reperfusion (IR) injury inevitably due to the natural characteristics of DCD donors with hypotension and cardiac arrest. This kind of warm IR injury could increase the risk of tumor recurrence after liver transplantation induced by accumulation of endothelial progenitor cells [9], T cells [10], and other immune cells. Neutrophils, which were the first-line responders to IR injury [11], had potential in promoting liver metastases after surgical stress [12]. Although the recruitment of neutrophils towards targeted organs was associated with formation of pre-metastatic niches [13], its exact role in ischemic grafts for transplantation remains under debate. Additionally, the existence of neutrophil heterogeneity, which was first reported by Fridlender et al in 2009 [14], has been investigated in many diseases including inflammation [15] and malignancies [16]. Neutrophils can be classified into different phenotype according to the surface receptors and functional characteristics, which could be altered by cytokine exposure [17]. On the other hand, it is revealed that neutrophils were significantly increased in the pre-transferred lungs before tumor cell arrival [18], which means neutrophils recruited by specific pathological condition could created a suitable premetastatic environment for tumor metastasis or recurrence. So we wonder whether warm IR injury could induce alteration of neutrophils polarization which probably forms a pre-metastatic microenvironment that promote HCC recurrence after liver transplantation.

Poly (ADP-ribose) polymerase (PARP), which is involved in physiological cellular processes, has been shown to aggravate liver damage during hepatic IR injury. PARP-1 inhibition, however, could attenuate microvascular injury and reduce the numbers of adherent leukocytes induced by hepatic IR injury [19]. To date, the influence of PARP-1 inhibition on neutrophils activities and HCC recurrence after liver transplantation has not been reported. This study aimed to explore whether PARP-1 inhibition could reduce the risk of HCC recurrence after liver transplantation and to elucidate the potential mechanisms with neutrophils.

## RESULTS

### PARP-1 increased the risk of IR-induced HCC recurrence after liver transplantation

**Figure 1:**
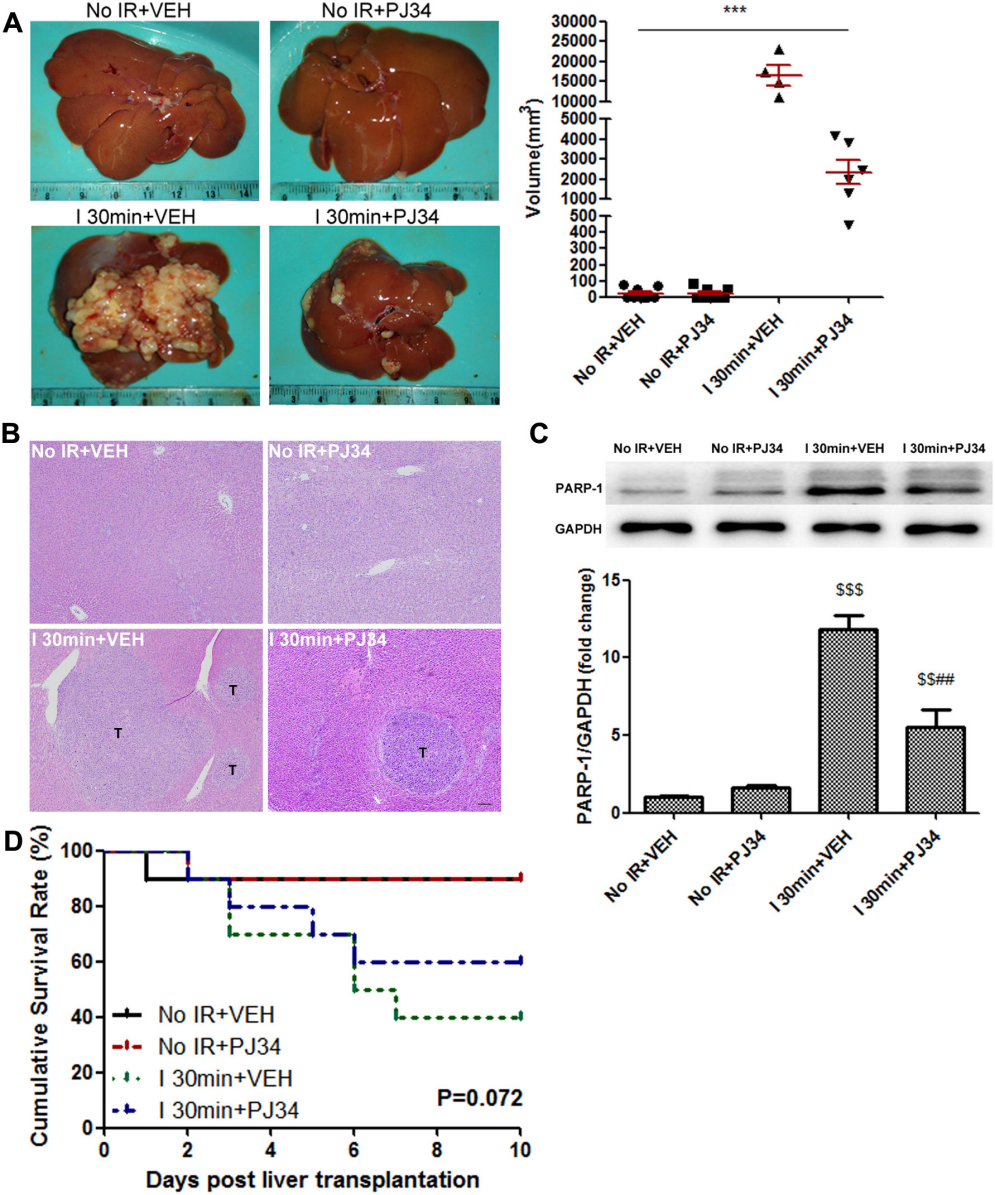
PARP-1 increased the risk of IR-induced HCC recurrence after rat liver transplantation **(A)** The recipient rats with 30min warm ischemic grafts were characterized by enhanced HCC recurrence at day 10 after liver transplantation. PARP-1 inhibition resulted in a significant decrease in the volume of tumors between I 30min groups. **(B)** The HE staining of liver and hepatic tumor (bar=100μm). **(C)** Liver protein expression of PARP-1 was up-regulated by hepatic warm IR injury and inhibited by PJ34. **(D)** Kaplan-Meier survival curve of rats in different groups (n=10 per group). mean ± SEM are shown.*p<0.05, **p<0.01, ***p<0.001. ^$^p<0.05, ^$$^p<0.01, ^$$$^p<0.001 compared to the No IR+VEH group. ^#^p<0.05, ^##^p<0.01, ^###^p<0.001 compared to the I 30min+VEH group; Data were analyzed by either Student’s t-test or by ANOVA as appropriate followed by Bonferroni post-tests.

To study the effect of warm IR injury on HCC recurrence after liver transplantation, we prepared ischemic donor livers and further conducted rat orthotopic liver transplantation. CBRH-7919, a kind of rat HCC cells, was injected via the portal vein after transplantation. As shown in Figure 1A, donor warm ischemia led to an increased risk of post-transplant HCC recurrence and growth 10 days after liver transplantation in Ischemia 30min (I 30min) group treated by vehicle (VEH). At the same time, HE staining of the liver grafts demonstrated the same trend on recurrence (Figure 1B). We also noticed that these process were accompanied by PARP-1 up-regulation in hepatic tissues due to IR injury (Figure 1C). Considering that PARP-1 promoted apoptosis/necrosis during IR injury [20] and high PARP-1 expression was associated with tumor malignant potential [21], we adopted the PARP-1 inhibitor PJ34 to explore its role in HCC recurrence after liver transplantation. PARP-1 was stably inhibited in liver grafts by pre- and post-operation daily injection of PJ34 (Figure 1C). Strikingly, we found that PJ34 markedly inhibited HCC recurrence in donor livers after liver transplantation in I 30min group (Figure 1A, 1B). Besides, only 4 of 10 rats survived in I 30min group while there were 9 rats in the No IR group survived. By contrast, PJ34 improved survival rates, to some extent, in I 30min group (Figure 1D, P=0.072). This suggested that PARP-1 might promote HCC recurrence after liver transplantation, highlighting the possibility to inhibit PARP-1 for preventing HCC recurrence.

### PARP-1 inhibition decreased the susceptibility of the liver to recurrence after IR injury

**Figure 2:**
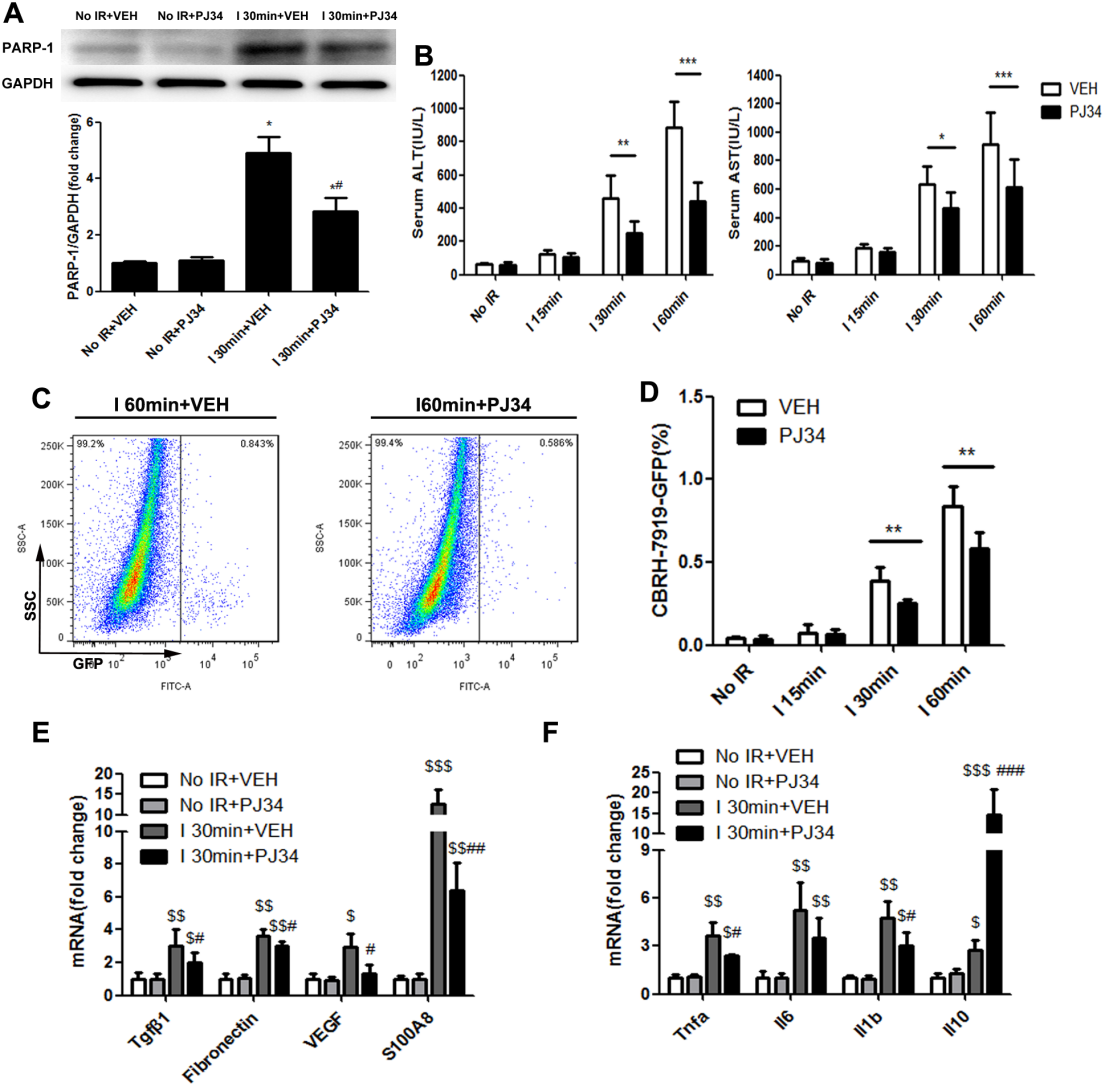
PARP-1 inhibition decresed the susceptibility of the liver to recurrence after IR injury in mice **(A)** Up-regulation of PARP-1 expression induced by hepatic warm IR injury was reduced after PJ34 administration. **(B)** PJ34 reduced serum ALT and AST, indicators for liver damage, after hepatic warm IR injury. **(C)** At 12h after reperfusion, flow cytometry was used to detect the CBRH-7919-GFP tumor cells implantation rates in single-cell suspension from ischemic liver lobes treated by VEH and PJ34. **(D)** PJ34 significantly reduced the engraftment rates of tumor cells in I 30min and I 60min group (n=6-8 per group). **(E, F)** Hepatic relative gene expression of pre-metastatic factors and cytokines was evaluated. mean ± SEM are shown. *p<0.05, **p<0.01, ***p<0.001. ^$^p<0.05, ^$$^p<0.01, ^$$$^p<0.001 compared to the No IR+VEH group. ^#^p<0.05, ^##^p<0.01, ^###^p<0.001 compared to the I 30min+VEH group; Data were analyzed by either Student’s t-test or by ANOVA as appropriate followed by Bonferroni post-tests.

We tried to mimic the transplantation setting in which, at the time of recipient liver removal in HCC patients, there are disseminated tumor cells in the circulation that might become the “seed” for recurrence. Therefore, intrasplenic injection of GFP-tagged hepatoma cells and circulation for 10min before warm hepatic IR was conducted in mice [22]. According to the hepatic lesion alterations embodied in serum hepatic enzymes at different reperfusion time (Supplementary Figure 1B, 1C), we chose the hepatic repair phase (reperfusion time point: 12h) for further studies to elucidate the role of PARP-1 up-regulation during warm IR in tumor recurrence. Mice hepatic PARP-1 expression was increased after warm IR as well (Supplementary Figure 1A). We examined the engraftment rates of metastatic tumor cell arrival in liver of mice through flow cytometry after 12h reperfusion. As a result, increasing number of CBRH-7919-GFP was detected in ischemic hepatic lobe and the longer the warm ischemic time, the higher the proportion of invading tumor cells was (Figure 2C, 2D). However, PJ34 treatment inhibited PARP-1(Figure 2A), improved the hepatic enzymes (Figure 2B), and decreased the engraftment rates of tumor cells (Figure 2C, 2D) compared to mice which suffered from the same ischemic time without PARP-1 inhibition. We also detected the tumor burden in the same way with syngeneic mouse HCC cell line (Hepa1-6-GFP) and got the similar results (Supplementary Figure 1D, 1E). It's worth noting that GFP+ events were free cells in the liver instead of being phagocyted by macrophages cells (Supplementary Figure 1F). It seems that PARP-1 inhibition was a feasible way to decrease tumor cells homing to liver after IR injury. Furthermore, we investigated whether the CBRH-7919 intrinsic capability to metastasize was inhibited by PJ34. Pre-incubation with PJ34 did not inhibit the invasion of CBRH-7919 *in vitro* and the homing ability to form HCC recurrence *in vivo* (Supplementary Figure 2A, 2B). These results above revealed that PARP-1 inhibition did not directly work on tumor cells to decrease their malignant potential. On the other hand, we explored the susceptibility of the liver by analyzing the hepatic markers which were reported to be associated with the formation of proliferative environment [23]. Downregulation of matrix metalloproteinase 9(MMP9) and vascular endothelial growth factor (VEGF) A, which were up-regulated by IR injury and had potential in facilitating tumor cells invasion and angiogenesis, were confirmed in mice liver pretreated with PJ34(Supplementary Figure 2C, 2D). Additionally, hepatic expression of the pre-metastatic milieu related factors and cytokines was significantly attenuated by PJ34 as compared to mice without PARP-1 inhibition except *Il10* (Figure 2E, 2F). Thus, we supposed that PARP-1 inhibition decreased the risk of HCC recurrence after liver transplantation through reduction of hepatic susceptibility to recurrence.

### CXCL1/CXCR2 axis was crucial for PARP-1-induced hepatic susceptibility to recurrence after IR injury

**Figure 3:**
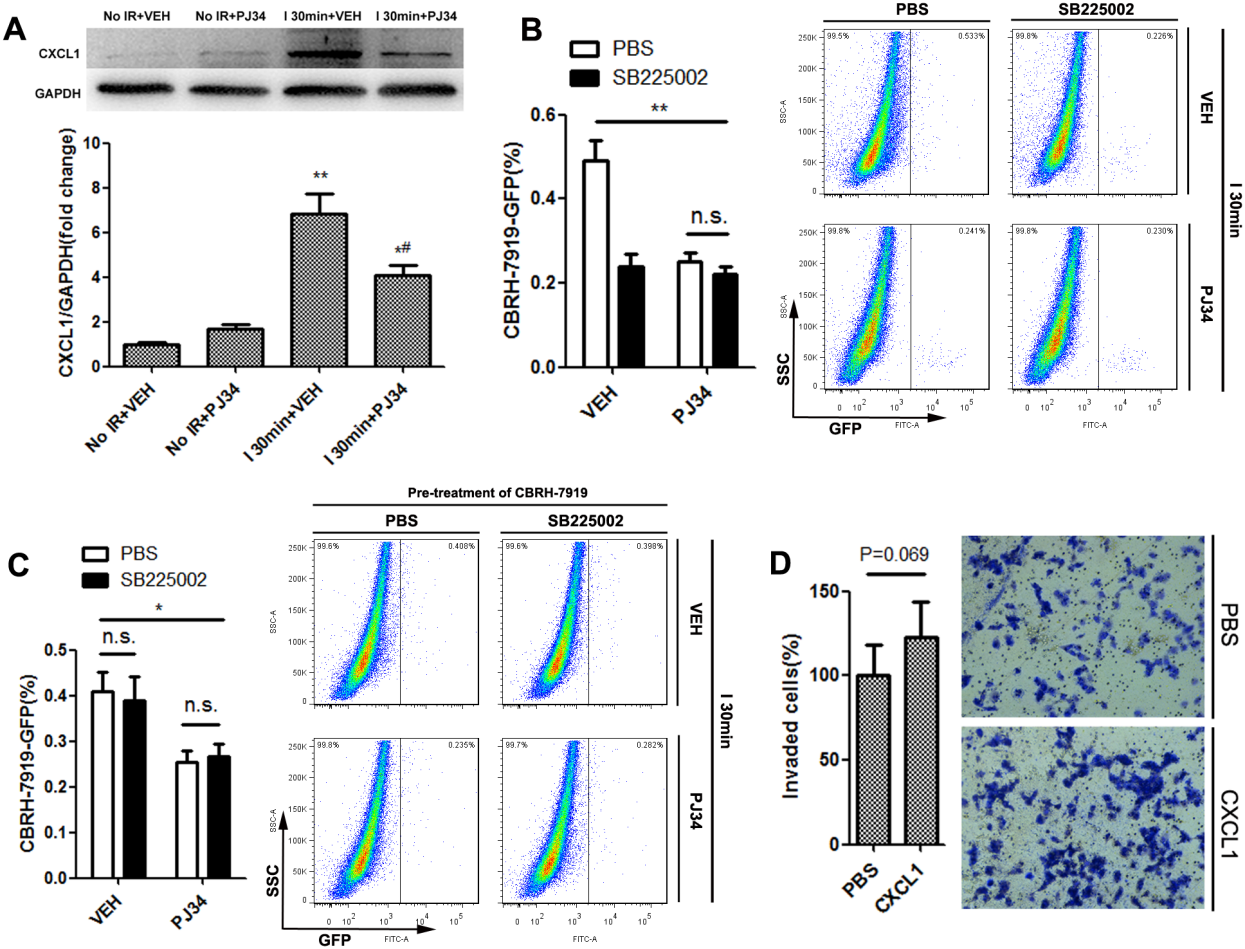
CXCL1/CXCR2 axis was crucial for PARP-1-induced hepatic susceptibility to recurrence after IR injury in mice **(A)** CXCL1 changes induced by VEH or PJ34 after liver IR injury were confirmed through western blot. **(B)** Mice in I 30min group were administrated with CXCR2-inhibitor SB225002 or PBS prior to hepatic IR injury treated by PJ34 or VEH. Flow cytometry was used to quantify recurrence burden. (n=6-8 per group) **(C)** CBRH-7919-GFP cells were incubated with SB225002 or PBS prior to splenic injection in I 30min group. Flow cytometry was used to quantify recurrence burden. (n=6-8 per group) **(D)** *In vitro* transwell invasion of CBRH-7919 towards CXCL1. mean ± SEM are shown. *p<0.05, **p<0.01, ***p<0.001. n.s., not significant; Data were analyzed by either Student’s t-test or by ANOVA as appropriate followed by Bonferroni post-tests.

Liver IR injury is a dynamic process involving immune insult with disturbances of multiple chemokines and growth factors [24]. It is possible that PARP-1 up-regulation after liver IR injury promotes recurrence by recruiting circulation tumor cells through disordered chemokines. Therefore, we compared chemokines levels secreted in different groups of mice using Chemokine Antibody Array. Among the 25 chemokines tested in this study, CXCL1 was the one which was significantly increased after hepatic IR injury and decreased when PARP-1 was inhibited at the same time (Supplementary Figure 3A, 3B). This alteration was further confirmed at both the transcript and protein levels (Supplementary Figure 3C, Figure 3A). CXCL1 levels in the plasma, however, remained unchanged upon inhibition of PARP-1 after liver IR (Supplementary Figure 3D). Since CXCL1 bind to CXCR2, we next used SB225002, a selective CXCR2 antagonist, in order to elucidate the functional relevance of CXCL1 in the susceptible microenvironment induced by PARP-1 after liver IR. We found that administration of SB225002 in mice could significantly reduce PARP-1-promoted invading of tumor cells to liver after IR injury (Figure 3B). However, pre-incubation of CBRH-7919-GFP with SB225002 did not reduce its homing ability to liver after IR injury (Figure 3C). And also, CXCL1 stimulation for CBRH-7919-GFP failed to significantly enhance the migration and invasion *in vitro* (Figure 3D). It seems that CXCL1/CXCR2 signaling activated hepatic microenvironment and increased the susceptibility to recurrence rather than directly chemotactical attraction of tumor cells towards the liver.

### CXCL1-mediated neutrophils recruitment was required for PARP-1-induced hepatic susceptibility of the liver to recurrence after IR injury

**Figure 4:**
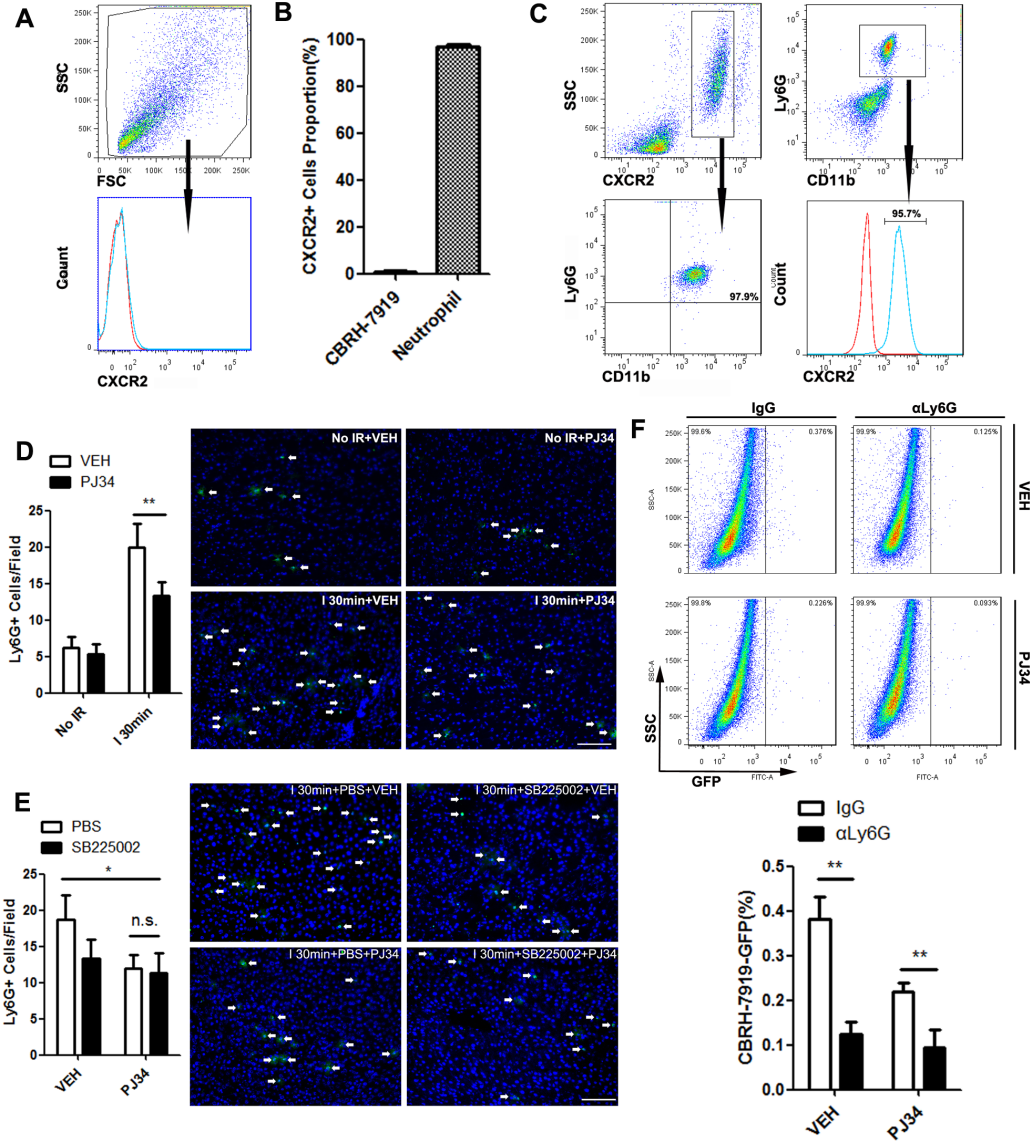
CXCL1-mediated neutrophils recruitment was required for PARP-1-induced hepatic susceptibility to recurrence after IR injury in mice **(A)** CXCR2 surface levels of CBRH-7919 were detected by flow cytometry. **(B)** Quantitative data for percentage of CXCR2-positive cells in total CBRH-7919 and circulating neutrophils. **(C)** Peripheral blood cells from mice in I 30min group were analyzed by flow cytometry. **(D)** Representative micrographs of neutrophil infiltration with or without PJ34 treatment in mice liver after warm IR injury (n=6-8 per group, bar=100μm). **(E)** Representative images of neutrophilic cells infiltration into the 30min ischemic liver lobes in PBS- and SB225002-treated mice with or without PARP-1 inhibition (n=6-8 per group, bar=100μm). **(F)** Hepatic recurrence burden with CBRH-7919-GFP after anti-Ly6G antibody injection was analyzed by flow cytometry in I 30min groups with or without PARP-1 inhibition. (n=6-8 per group) mean ± SEM are shown. *p<0.05, **p<0.01, ***p<0.001. n.s., not significant; Data were analyzed by either Student’s t-test or by ANOVA as appropriate followed by Bonferroni post-tests.

Since CXCR2 is a most important target for CXCL1, we further explored its expression during this pathophysiological process. Intriguingly, CBRH-7919-GFP expressed very low levels of CXCR2 (Figure 4A, 4B), which could explain its bluntness towards CXCL1 chemotactic effect. Then how does recurrence-favorable microenvironment form before tumor cells infiltration? Previous studies suggested that bone marrow-derived cells extensively involved in this process [25]. So we detected the cells in the blood of liver IR mice (ischemic time=30min, reperfusion time=12h) and found that nearly all the CXCR2-positive cells were neutrophils, and also the vast majority of neutrophils could expressed CXCR2 (Figure 4B, 4C). Next, the alteration of hepatic neutrophil number in hepatic IR mice (ischemic time=30min) was analyzed. Immunofluorescent assay of liver tissue showed that increased neutrophils infiltration caused by IR injury could be down-regulated by PARP-1 inhibition (Figure 4D). In addition, administration of SB225002 prevented PARP-1-induced recruitment of neutrophils to the liver after IR (Figure 4E). To characterize the indispensability of neutrophils recruitment for PARP-1-induced tumor cells invading, we next systemically depleted neutrophils and found that the engraftment rates of CBRH-7919-GFP to the liver were decreased in spite of hepatic IR injury (Figure 4F). We also performed an experiment in the same way with a syngeneic mouse HCC cell line after ischemia. The injection of Ly6G antibody could reduce the hepatic Hepa1-6-GPF tumor burden as well (Supplementary Figure 4A). *In vitro*, CBRH-7919 co-cultured with fresh isolated neutrophils showed enhanced migration which was associated with epithelial-mesenchymal transition (EMT) (Supplementary Figure 4B, 4C). All these above revealed that neutrophils recruited by CXCL1 increased the hepatic susceptibility to recurrence after IR injury.

### Warm hepatic IR injury converted neutrophils into a proangiogenic type in liver

**Figure 5:**
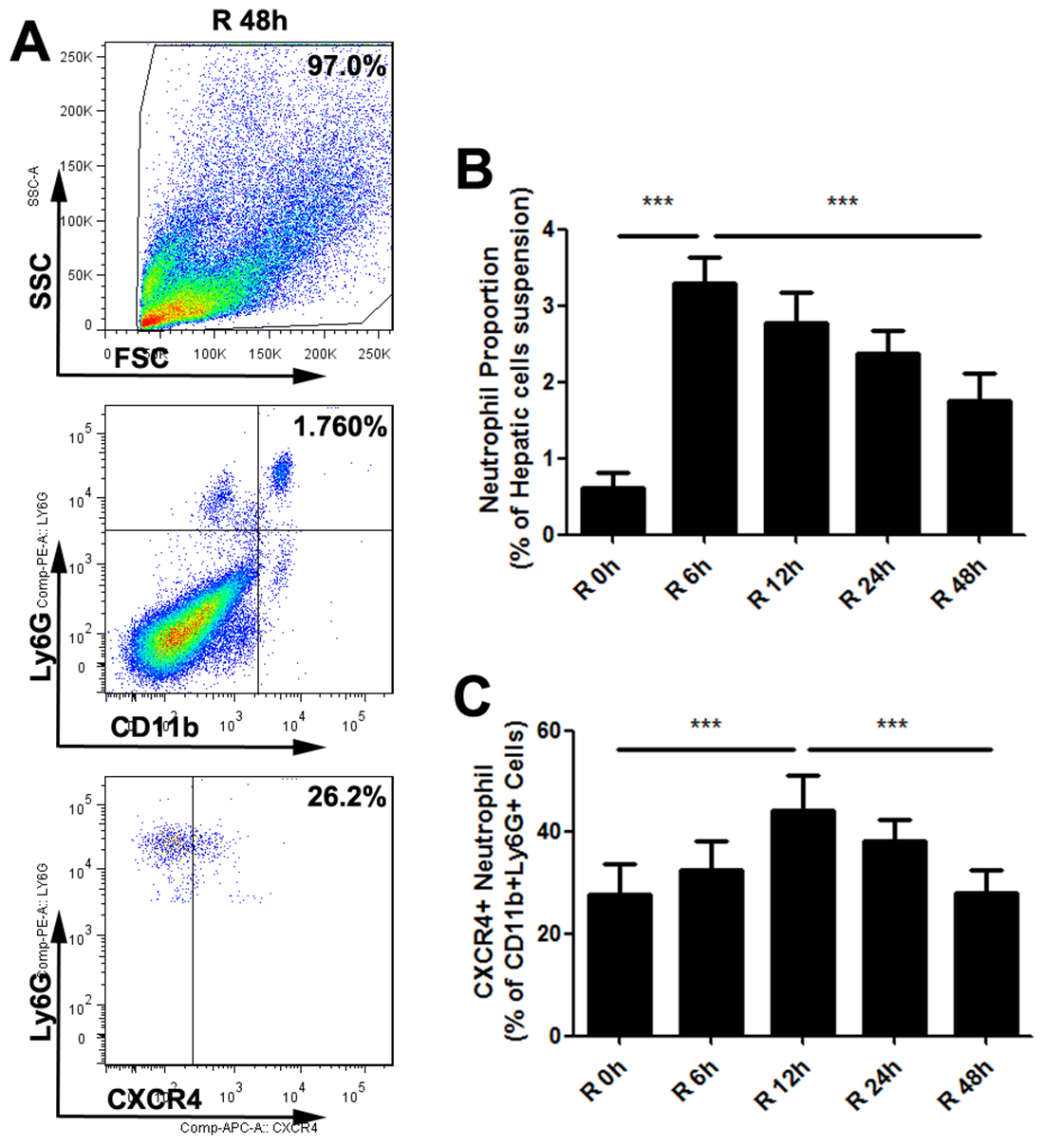
Warm hepatic IR injury altered the proangiogenic neutrophil proportion in mice liver Representative flow cytometry plots **(A)** for one time point (Reperfusion time=48h) elucidated gating strategy to identify neutrophil proangiogenic phenotype. The percentages of neutrophils in hepatic single-cell suspension **(B)** and the proangiogenic neutrophil proportion **(C)** at different reperfusion time were displayed in histogram (n=6-8 per group). mean ± SEM are shown. *p<0.05, **p<0.01, ***p<0.001; Data were analyzed by either Student’s t-test or by ANOVA as appropriate followed by Bonferroni post-tests.

As indicated above, warm hepatic IR led to the accumulation of neutrophils in liver which is consistent with previous data [26]. However, the phenotypic characterization of these neutrophils present in liver after IR has not been reported yet. To that aim, we quantified the number of proangiogenic neutrophils in the liver after liver IR injury. Christoffersson G, et al demonstrated that CD11b+Ly6G+ neutrophils which expressed high levels of the CXCR4 in surface could deliver large amounts of MMP9 and further facilitate rapid angiogenesis and restoration of perfusion in the hypoxic microenvironments after islet transplantation [27]. Therefore, we defined proangiogenic neutrophils as CD11b+Ly6G+CXCR4+. Next we evaluated neutrophil phenotype using flow cytometry in mice liver which suffered from different reperfusion time after 30min hepatic ischemia. The liver neutrophil numbers peaked at reperfusion time 6h and decreased after that (Figure 5A, 5B). However, the percentage of proangiogenic neutrophils significantly increased post-IR and peaked at reperfusion time 12h (Figure 5A, 5C). High proportion of proangiogenic neutrophils could promote phagocytosis of apoptotic cells, resolution of inflammation, and repair of ischemic foci [28], but it seems to facilitate the circulating tumor cell implanting cascade at the same time. Limiting excessive immune restoration activity may be a promising idea to reduce tumor recurrence or metastasis after pathophysiologic stress such as IR.

### PAPR-1 inhibition decreased the hepatic proangiogenic neutrophil proportion up-regulated by liver IR injury

**Figure 6:**
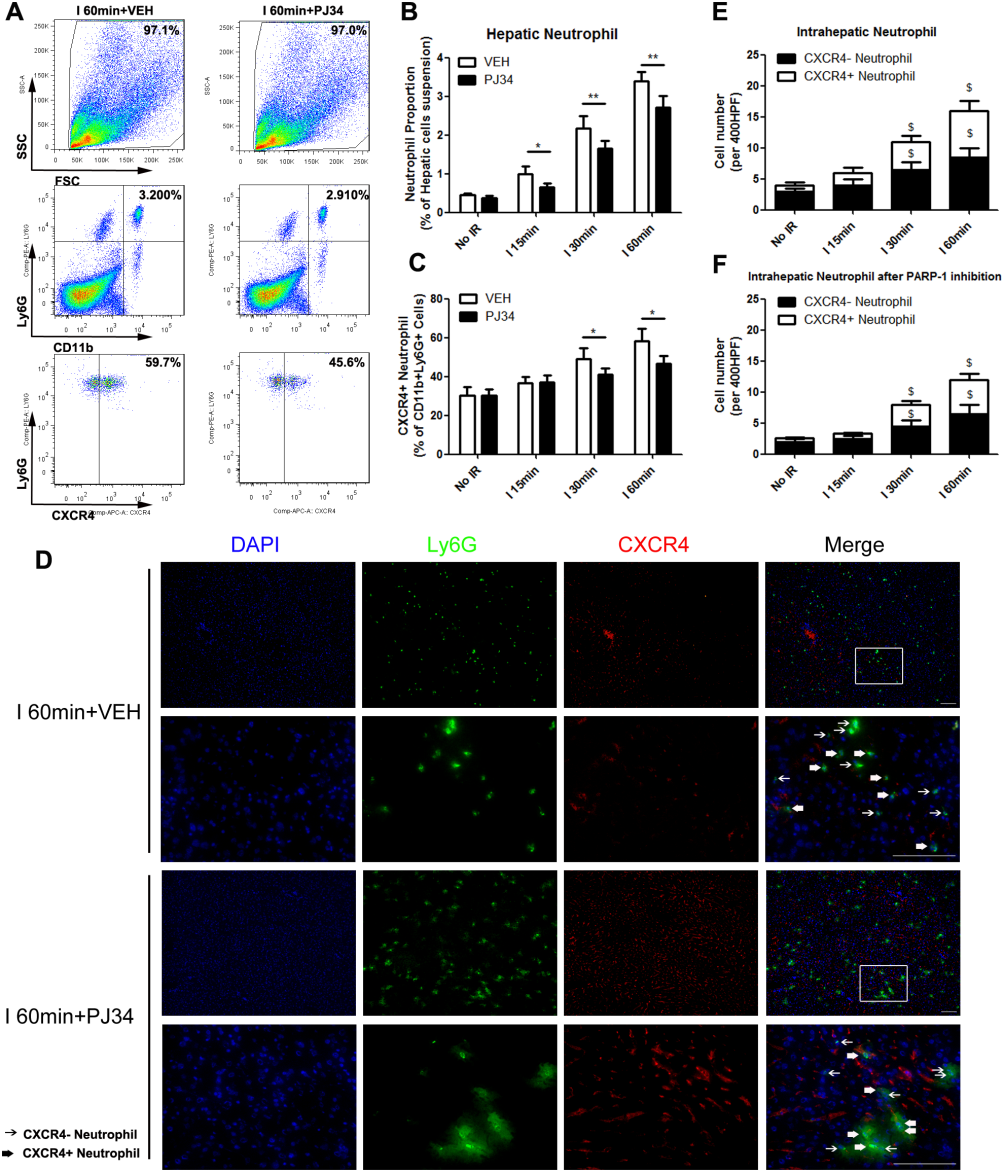
PAPR-1 inhibition decreased the hepatic proangiogenic neutrophil proportion up-regulated by liver IR injury in mice Hepatic neutrophils in mice with different warm ischemic time from VEH- or PJ34-treated **(A)** groups were analyzed by flow cytometry at 12 hours after reperfusion. Quantification of hepatic neutrophils **(B)** and CXCR4+ neutrophils **(C)** in hepatic IR mice after VEH or PJ34 treatment was presented. Representative double immunofluorescence of Ly6G (Green) and CXCR4(Red) for proangiogenic neutrophils located in the VEH- or PJ34-treated ischemic liver lobe after 12h reperfusion **(D)** was shown (bar=100μm, n=6-8 per group). The immunofluorescence numbers of neutrophils that colocalized with CXCR4 12h after reperfusion in VEH- **(E)** or PJ34-treated **(F)** mice were counted manually in three random high-powered fields (HPF). mean ± SEM are shown. *p<0.05, **p<0.01. ^$^p<0.05, compared to the No IR group; Data were analyzed by either Student’s t-test or by ANOVA as appropriate followed by Bonferroni post-tests.

In order to deeply illustrate the association between hepatic IR injury and liver proangiogenic neutrophil proportion, we quantified the number of proangiogenic neutrophils as CD11b+Ly6G+CXCR4+ cells in mice liver with different ischemic time after 12h reperfusion. To explore the role of PARP-1 during this process, PJ34 was administrated for PARP-1-inhibition groups and VEH was for others. Firstly, we observed the alteration in groups without PARP-1 inhibition. In the group without warm IR, 30.4±4.1% of infiltrated neutrophils were proangiogenic. Hepatic proangiogenic neutrophil proportion were significantly increased with the prolongation of liver ischemic time and up to 58.4±6.1% in group with ischemia time 60min. In this process, total hepatic neutrophils number was elevated by IR injury as well (Figure 6A-6C). These trends were also confirmed by immunofluorescence semi-quantitative analysis of Ly6G+CXCR4+ cells. (Figure 6D-6F). Secondly, mice injected with PJ34 were analyzed. The rising trend of total neutrophils and proangiogenic neutrophil numbers in liver induced by IR was observed in PARP-1 inhibition groups. However, the hepatic proangiogenic neutrophil proportion was remarkably lower in I 30min group and 60min group when PARP-1 was inhibited (Figure 6A-6C). What’s more, neutrophil recruitment to liver was also reduced in all groups with hepatic IR injury by PJ34. The immunofluorescence presented the similar phenomena as well (Figure 6D-6F). These results indicated that PARP-1 inhibition decreased the percentage of proangiogenic neutrophil in the liver after IR injury.

### PARP-1 had no influence on the polarization of neutrophils outside of liver milieus

**Figure 7:**
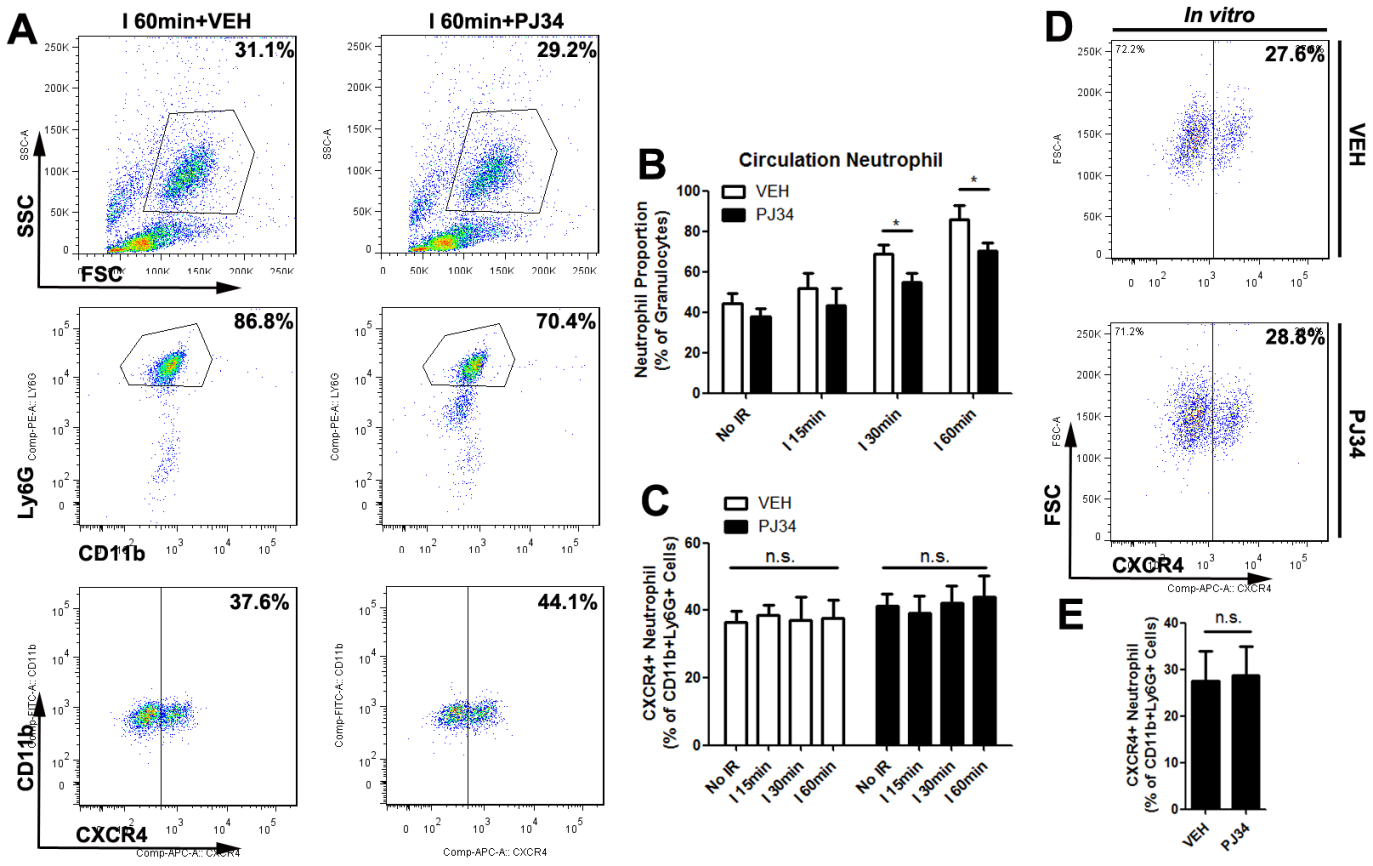
PARP-1 had no influence on the polarization of neutrophils outside of mice liver milieus Blood cells were stained for 3-color flow cytometry with monoclonal antibodies against CD11b, Ly6G, and CXCR4 12 hours after liver reperfusion with VEH or PJ34 treatment **(A)**. Quantification of circulation neutrophils **(B)** and CXCR4+ neutrophils **(C)** in hepatic IR mice after VEH or PJ34 treatment was presented (n=6-8 per group). In addition, PJ34 (1μg/ml for 12h) failed to polarize isolated neutrophils phenotype *in vitro* **(D, E)**. mean ± SEM are shown. *p<0.05. n.s., not significant; Data were analyzed by either Student’s t-test or by ANOVA as appropriate followed by Bonferroni post-tests.

The reason why PARP-1 inhibition lowered the hepatic proangiogenic neutrophil proportion was indistinct. There may be two possible mechanisms for this. First is that PARP-1 inhibition specifically suppressed circulation proangiogenic recruiting into liver after IR. In order to prove it, we determined the total number of neutrophils and proangiogenic neutrophils in peripheral blood by flow cytometry 12h after reperfusion (Figure 7A-7C). We found that the hepatic IR didn’t significantly influence the circulation proangiogenic proportion. Although PJ34 reduced the percentage of circulation neutrophils in ischemia 30min group and 60min group, there were almost no changes in the proangiogenic neutrophil proportion as well. PARP-1 inhibition seemed to prevent neutrophils shifting to a proangiogenic phenotype in liver after hepatic IR injury though PJ34 didn’t change the fresh isolated neutrophil phenotype *in vitro*. (Figure 7D-7E) Therefore, the possible mechanism of hepatic neutrophil phenotype shifting was probably not about differential recruitment on proangiogenic neutrophils but about programming towards proangiogenic neutrophils only in hepatic immune microenvironment.

## DISCUSSION

**Figure 8:**
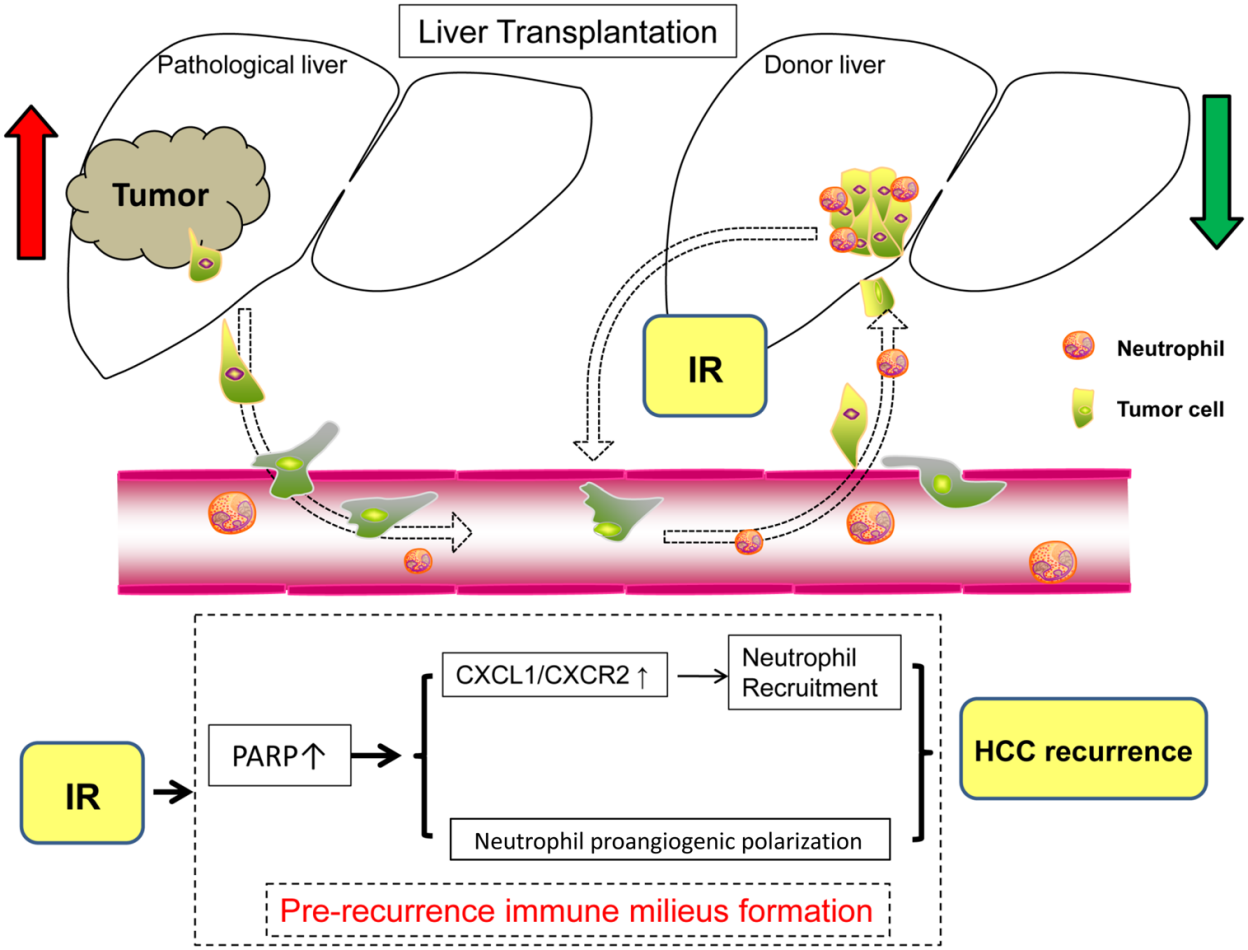
Schematic diagram for the mechanisms of PARP-1 in the promotion of HCC recurrence after liver transplantation through IR-mediated neutrophils recruitment and polarization

Post-liver transplantation HCC recurrence has always been a critical issue for transplant surgery. Liver grafts subjected to inevitable IR injury are related to an increased risk of HCC recurrence and growth after liver transplantation [8], of which the precise mechanisms haven’t been clearly illustrated. Factors such as hypoxia, ischemia and inflammation could remodel the grafts’ microenvironment and promote the mobilization of bone marrow progenitors to form premetastastic niches [29]. Here, we show for the first time that PARP-1 up-regulated by hepatic IR injury could increase the hepatic susceptibility to post-liver transplantation HCC recurrence through regulation of neutrophil recruitment and phenotype shifting (Figure 8).

Intriguingly, PARP-1 activity is involved in IR injury and tumor progress, both of which promote HCC recurrence after liver transplantation. Under conditions of IR, activated PARP consumes NAD+ in order to repair damaged DNA, but its hyperactivation promotes cell death by causing ATP depletion, amplifying oxidant stress and further decline in mitochondrial function, which aggravate IR injury [30]. This vicious circle can be broken by inhibiting PARP because the PARP inhibitor offers protection against IR injury for brain [31], lung [32], and kidney [33]. It seems that amelioration of hepatic IR injury via PARP inhibition could be a promising method to reduce the risk of HCC recurrence after liver transplantation. As to tumor progress, PARP-1 was revealed to be overexpressed in numerous malignant tumors and associated with invasiveness and poor prognosis. PARP inhibitors are currently treated as potential anticarcinogen in clinical trials for ovarian cancer [34], breast cancer [35], and so on although its direct effect on HCC is still elusive. In this study, PJ34-mediated protection against IR-induced hepatic injury could reduce the plasma ALT and AST, arrest the induction of inflammation genes, and decrease the infiltration of neutrophils. However, the migration and engraftment of tumor cells with PARP-1 inhibitor pretreatment didn’t significantly altered *in vivo* or *in vitro*, suggesting that PARP-1 inhibition is an important protector of HCC recurrence after IR by preventing the formation of susceptible hepatic milieus rather than decreasing the degree of malignant in HCC.

Since PJ34 failed to directly reduce CBRH-7919 migration in our study, it is supposed that hepatic PARP-1 up-regulation after IR enhanced HCC recurrence through regulation of other pathways such as chemokines. Chemokines are identified as regulators of cell mobility which are important for metastasis by modulating the motility and survival of spreading cancer seeds or by activating immune and stromal cells to promote metastasis [36]. Here, we sought out CXCL1 as the one which was decreased when PARP-1 was inhibited after hepatic IR injury in the Chemokine Antibody Array. Consistently, blockade of CXCR2 systemically in mice instead of tumor cells only could reduce engraftment rates of tumor cells homing to liver after IR injury, indicating that CXCL1/CXCR2 signaling is critical in PARP-1-induced susceptibility of the liver to recurrence. Neutrophils, which have potentials in promoting tumor growth and metastasis [37], were recruited by CXCL1/CXCR2 axis after liver IR injury and took part in facilitating tumor recurrence as proven by neutrophil-depletion-mediated protection against tumor cells invading. These pro-tumorigenic effects of PARP-1 required neutrophils. Immune cells infiltrating into a specific organ before tumor cells arrival could initiate a metastasis cascade in a tumor-bearing host. Nevertheless, these pioneer immune cells are mobilized by inflammation or immune-disorder in that specific organ caused by the distant primary tumor [18]. With regard to liver transplantation for HCC candidates, inflammation and immune-disorder in donor grafts caused by IR-based stress could mimic the pre-metastatic milieu resulted from primary HCC even though recipient HCC liver has been removed. As an important pathogenic factor in this process, neutrophils participated in the inflammation outbreak and then immune repair through phenotype alteration probably. After hepatic ischemia, the early phase of reperfusion (within 6h) caused hepatocellular apoptosis, necrosis and sinusoids narrowing which led to entrapment of platelets and neutrophils [38]. These neutrophils extravasated into the parenchyma and played a cytotoxic role in a pro-inflammatory method [39]. And over time, the percentage of anti-inflammatory neutrophils increased in order to resolve inflammation and repair wound which, however, may contribute to tumor cells planting, growth and further angiogenesis if all of these happened to a tumor-bearing host.

In this study, enrichment of proangiogenic neutrophils in liver after IR injury was probably induced by PARP-1-mediated local phenotype shifting because the percentage of circulating proangiogenic neutrophils was not changed. And we failed to change neutrophil phenotype with PJ34 *in vitro*, indicating that there must be intermediaries between PARP-1 and neutrophil phenotype shifting. Since PARP-1 suppresses regulatory T cells generation, controls TGF-β receptors on T cells, and facilitates T helper (Th) cells differentiation [40], we hypothesize that PARP-1 inhibition could influence the Th cells cytokines balance which further promotes neutrophil phenotype shifting. For example, PARP-14 promoted Th2 differentiation by regulating IL-4-dependent transcription [41]. Additionally, olaparib, a kind of PARP inhibitor, protected mice against asthma by suppression of Th2 cytokines such as IL-4 [42]. IL-4 is a strong stimulus for proangiogenic neutrophil shifting and we believe that the mechanism for proangiogenic neutrophil polarization after liver IR injury is closely related to PARP-1-mediated Th2 differentiation and Th2 cytokines preferential expression. These hypotheses need to be proven in further studies.

Actually, HCC recurrence after liver transplantation is a complex pathophysiological process which could be affected by many factors. Recipient conditions, such as tumor characteristics, hepatitis virus status, obesity, and advanced age, are usually out of control. For example, Li et al reported that recipient high HBV DNA levels before transplantation are related to HCC recurrence after transplant [43]. So quality control for donor grafts seems to be more feasible. In recent years, the expanding of donor types is making IR injury increasingly emerging. Living donor grafts [44], DCD donors [7], and split grafts [45] are reported to be associated with tumor recurrence after liver transplantation due to IR injury. In this research, we pointed out PARP-1 as a promising target to protect against IR injury and HCC recurrence after liver transplantation at the same time. Since PARP inhibitor is a recently FDA-approved therapy for cancer, our research has clinical significance and could offer new approaches to prevent or even treat HCC recurrence after liver transplantation.

In conclusion, this study is the first time to identified PARP-1, which is up-regulated by hepatic IR injury, as a promoter of HCC recurrence after liver transplantation through creating a recurrence-susceptible milieu. PARP-1 enhanced CXCL1/CXCR2 axis after hepatic IR injury facilitates the recruitment of neutrophils which promote HCC recurrence after transplantation. In addition to increasing its quantity, PARP-1 also stimulated hepatic neutrophils proangiogenic polarization *in vivo*. Therefore, specifically inhibiting PARP-1 may represent novel therapeutic strategies for HCC candidates with liver transplantation by ameliorating liver IR injury induced neutrophils recruitment and proangiogenic polarization, and then prevent HCC recurrence.

## MATERIALS AND METHODS

### Animals

Experiments were conducted on male Wistar rats (200-250g) and male C57BL/6J mice (6 to 8 weeks old) which were purchased from the Laboratory Animal Center of the Affiliated Drum Tower Hospital of Nanjing University Medical School, and housed under specific pathogen-free conditions. The animal experiments were approved by the Institutional Animal Care and Use Committee of Nanjing University, China under the NIH Guide for the Care and Use of Laboratory Animals. All efforts were made to minimize suffering.

### Rat orthotopic liver transplantation

Isoflurane combined with chloral hydrate anesthesia followed by orthotopic rat liver transplantation was performed as previously described [8]. Donor livers were subjected to different ischemic time by clamping the liver pedicle and the infra-hepatic vena cava in order to mimic the cardiac arrest situation of DCD donors. They were described as No IR and I 30min with warm ischemia time for 0 and 30min respectively. Animal survival was determined after liver transplantation. The animals were monitored for 10 days, and survival was calculated using the Kaplan-Meier method. Then, recipient rats were sacrificed with the liver samples excised and preserved in 4% formalin or snap frozen in liquid nitrogen.

### Mouse hepatic IR injury

We induced 70% hepatic warm ischemia by clamping the hepatic artery, portal vein and bile duct branches to the left and median liver lobes, which in accordance with the previous studies [46]. Similar to the description above, the hepatic ischemic time determined the groups’ description as No IR, I 15min, I 30min, and I 60min. Blood and liver samples were collected at indicated time after reperfusion.

### Inhibitor administration

For liver transplantation models, the recipient rats were one day pre- and daily post-operation intraperitoneally (i.p.) administrated with either vehicle (VEH) or N-(6-Oxo-5.6-dihydrophenanthridin-2-yl)-(N,N-dimethylamino) acetamide hydrochloride (PJ34), the PARP-1 inhibitor obtained from Sigma-Aldrich, St. Louis MO(10 mg/kg). The donors received VEH or PJ34 intraperitoneally one day and one hour before transplantation. For hepatic IR injury models, mice in PARP-1 inhibition groups were treated with PJ34 at 10 mg/kg i.p. one day and one hour before the onset of ischemia. Where indicated, mice were administrated with 10 mg/kg SB225002 (Selleck) by i.p. injection, starting one day and one hour to prior to liver ischemia injury.

### Cell lines

A rat HCC cell line CBRH-7919 and mouse HCC cell line Hepa1-6 were originally obtained from from American Type Culture Collection (Manassas, VA, USA). CBRH-7919 was maintained in RPMI-1640 supplemented with 10% fetal bovine serum (Gibco, USA) at 37 °C in an atmosphere of 5% CO_2_ in air while Hepa1-6 was cultured in Dulbecco’s modified Eagle’s medium.

### HCC cell injection

For purpose of recreating the clinical condition of circulating tumor cells invading to the graft after liver transplantation, CBRH-7919(1×10^6^) suspended in 500μl PBS were injected via the portal vein after reperfusion in the rat model of liver transplantation. The hepatic tumor volume was calculated as previously described [47]. As to exploration on the relationship between IR injury and recurrence, 1×10^5^ GFP-targeted HCC cells in 100μl PBS were injected into the pulp of the anterior pole of the spleen [22]. Where indicated, CBRH-7919 was pre-incubated with PJ34(1μg/ml, 2h) or SB225002(1μg/ml, 2h) prior to splenic injection.

### Blood biochemistry

Blood samples were measured immediately using an automatic analyzer (Fuji, Tokyo, Japan) for alanine aminotransferase (ALT), aspartate aminotransferase (AST), as previously described [48].

### Cell suspension preparation and flow cytometry

Hepatic single-cell suspensions were made from mouse livers with gentleMACS Dissociator and mouse Liver Dissociation Kit (Miltenyi Biotec, Bergisch Gladbach, Germany) according to the manufacturer's instructions. Then the cell suspension was filtered through a 70-mm cell strainter. Peripheral blood from hepatic IR mice were incubated with FACS Lysing Solution (BD Pharmingen). For flow cytometry analysis, cell samples were stained with fluorescence-conjugated antibodies. Mouse FITC-anti-CD11b, PE-anti-Ly6G, and APC-anti-CXCR4 were purchased from BD Pharmingen. APC-anti-CXCR2 for mouse and rat were from R&D Systems (Minneapolis, MN, USA). Isotype-matched, fluorescently conjugated-antibodies of irrelevant specificity were used as controls. FlowJo Software (Tree Star Inc., Ashland, OR) was used for data analysis.

### Systemic neutrophil depletion

To deplete neutrophils, animals were i.p injected with 20 mg/kg of a specific antibody for neutrophil marker Ly6G (Biolegend) or control rabbit isotype as described previously [49].

### Peripheral blood neutrophil isolation

Peripheral blood neutrophils from liver IR mice were isolated as described previously [50]. We pooled three to five mice per sample to acquire a sufficient number of neutrophils for further experiments. These fresh isolated neutrophils were used for co-culturing with CBRH-7919 or being stimulated with PJ34(1μg/ml) for 12h to evaluate neutrophil phenotype by flow cytometry.

### Histological and immunohistochemical analysis

Paraffin liver sections (5 μm) were stained with hematoxylin and eosin (HE) for histological evaluation of HCC recurrence according to standard methods in routine pathology. As to neutrophils, tissue specimens cryosections (8 μm) were stained with Ly6G (R&D Systems) and CXCR4 (Abcam) primary antibodies as described previously [51]. Macrophages were labeled with F4-80(Abcam).

### Chemotactic cell invasion/migration

Costar Transwell Permeable Supports with 3μm pore size in the presence of Martrigel™-coating (Corning Inc.) were used for CBRH-7919 cells invasion assays [52]. Medium containing 100 ng/ml recombinant CXCL1 protein (Peprotech) was given in the lower chamber of the assay. For PJ34 pre-treatment assays, CBRH-7919 cells were incubated with PJ34 (1μg/ml, 2h) before being seeded into the upper chamber or injected into spleen. As to wound healing assays, CBRH-7919 were seeded in 6-well plates at a density of 2×10^5^ cells/well in RPMI Medium 1640 containing 10% fetal bovine serum (FBS) and then scraped at 80∼90% confluence. To assess wound closure, images were captured at 0, 24h time points in group co-cultured with neutrophils or control group [53].

### Quantitative real-time polymerase chain reaction (qRT-PCR)

Hepatic RNA was extracted from snap-frozen liver with the RNeay Midi Kit (Qiagen). Reverse transcription and qRT-PCR were performed as described previously [54]. Primers used for amplification were reported in Supplementary Table 1. Experiments were performed in triplicate and the values were normalized to GAPDH.

### Western blot analysis

Western blot was performed as described previously [48]. The blots were incubated overnight with primary anti-PARP-1 antibody (Abcam), anti-CXCL1 antibody (Abcam), anti-MMP9 antibody (Abcam), anti-VEGFA antibody (Abcam), anti-E-cardherin antibody (Abcam), anti-Vimentin antibody (Abcam), anti-Snail1 antibody (Abcam), anti-Slug antibody (Abcam), anti-Twist antibody (Abcam), and anti-GAPDH antibody (Abcam).

### Chemokine antibody array

A Mouse Chemokine Antibody Array (RayBiotech, Inc, Norcross, GA, USA) was used according to the manufacturer's instructions. The images were quantified using the ImageJ software. The intensities were normalized to internal positive controls for comparison.

### Enzyme-linked immunosorbent assay (ELISA)

The levels of CXCL1(Abcam) in mouse serum were analyzed using commercially available ELISA kits according to the manufacturer's instructions. The activities were expressed as fold change compared to the control group as described [55].

### Statistical analysis

Statistical analysis was performed with Graphpad Prism 5.0 and SPSS version 19.0, and data were expressed as mean± SEM for the indicated number of experiments. Normally distributed data were tested by Student’s t test. Where the normal distribution test failed, the Mann-Whitney test was applied. Differences between multiple groups were evaluated for significance using a one-way ANOVA combined with Bonferroni's post hoc test. P<0.05 was considered statistically significant.

## SUPPLEMENTARY MATERIALS FIGURES AND TABLE



## Abbreviations

PARP-1: poly (ADP-ribose) polymerase 1
IR: ischemia and reperfusion
HCC: hepatocellular carcinoma
DCD: donation after cardiac death
MMP9: matrix metalloproteinase-9
VEGF: vascular endothelial growth factor
EMT: epithelial-mesenchymal transition
VEH: vehicle
Th cells: T helper cells
ALT: alanine aminotransferase
AST: aspartate aminotransferase
HE: hematoxylin and eosin
FBS: fetal bovine serum
qRT-PCR: Quantitative Real-Time Polymerase Chain Reaction

## References

[1] Torre LA, Bray F, Siegel RL, Ferlay J, Lortet-Tieulent J, Jemal A (2015). Global cancer statistics, 2012. CA Cancer J Clin.

[2] Lafaro KJ, Demirjian AN, Pawlik TM (2015). Epidemiology of hepatocellular carcinoma. Surg Oncol Clin N Am.

[3] McGlynn KA, London WT (2005). Epidemiology and natural history of hepatocellular carcinoma. Best Pract Res Clin Gastroenterol.

[4] Song TJ, Ip EW, Fong Y (2004). Hepatocellular carcinoma: current surgical management. Gastroenterology.

[5] DeOliveira ML, Jassem W, Valente R, Khorsandi SE, Santori G, Prachalias A, Srinivasan P, Rela M, Heaton N (2011). Biliary complications after liver transplantation using grafts from donors after cardiac death: results from a matched control study in a single large volume center. Ann Surg.

[6] Hernandez-Alejandro R, Croome KP, Quan D, Mawardi M, Chandok N, Dale C, McAlister V, Levstik MA, Wall W, Marotta P (2011). Increased risk of severe recurrence of hepatitis C virus in liver transplant recipients of donation after cardiac death allografts. Transplantation.

[7] Croome KP, Wall W, Chandok N, Beck G, Marotta P, Hernandez-Alejandro R (2013). Inferior survival in liver transplant recipients with hepatocellular carcinoma receiving donation after cardiac death liver allografts. Liver Transpl.

[8] Oldani G, Crowe LA, Orci LA, Slits F, Rubbia-Brandt L, de Vito C, Morel P, Mentha G, Berney T, Vallee JP, Lacotte S, Toso C (2014). Pre-retrieval reperfusion decreases cancer recurrence after rat ischemic liver graft transplantation. J Hepatol.

[9] Li CX, Shao Y, Ng KT, Liu XB, Ling CC, Ma YY, Geng W, Fan ST, Lo CM, Man K (2012). FTY720 suppresses liver tumor metastasis by reducing the population of circulating endothelial progenitor cells. PLoS One.

[10] Li CX, Wong BL, Ling CC, Ma YY, Shao Y, Geng W, Qi X, Lau SH, Kwok SY, Wei N, Tzang FC, Ng KT, Liu XB, Lo CM, Man K (2014). A novel oxygen carrier “YQ23” suppresses the liver tumor metastasis by decreasing circulating endothelial progenitor cells and regulatory T cells. BMC Cancer.

[11] Peralta C, Jimenez-Castro MB, Gracia-Sancho J (2013). Hepatic ischemia and reperfusion injury: effects on the liver sinusoidal milieu. J Hepatol.

[12] Tohme S, Yazdani HO, Al-Khafaji AB, Chidi AP, Loughran P, Mowen K, Wang Y, Simmons RL, Huang H, Tsung A (2016). Neutrophil extracellular traps promote the development and progression of liver metastases after surgical stress. Cancer Res.

[13] Seubert B, Grunwald B, Kobuch J, Cui H, Schelter F, Schaten S, Siveke JT, Lim NH, Nagase H, Simonavicius N, Heikenwalder M, Reinheckel T, Sleeman JP (2015). Tissue inhibitor of metalloproteinases (TIMP)-1 creates a premetastatic niche in the liver through SDF-1/CXCR4-dependent neutrophil recruitment in mice. Hepatology.

[14] Fridlender ZG, Sun J, Kim S, Kapoor V, Cheng G, Ling L, Worthen GS, Albelda SM (2009). Polarization of tumor-associated neutrophil phenotype by TGF-beta: “N1” versus “N2” TAN. Cancer Cell.

[15] Cuartero MI, Ballesteros I, Moraga A, Nombela F, Vivancos J, Hamilton JA, Corbi AL, Lizasoain I, Moro MA (2013). N2 neutrophils, novel players in brain inflammation after stroke: modulation by the PPARgamma agonist rosiglitazone. Stroke.

[16] Sionov RV, Fridlender ZG, Granot Z (2015). The multifaceted roles neutrophils play in the tumor microenvironment. Cancer Microenviron.

[17] Mantovani A, Cassatella MA, Costantini C, Jaillon S (2011). Neutrophils in the activation and regulation of innate and adaptive immunity. Nat Rev Immunol.

[18] Yan HH, Pickup M, Pang Y, Gorska AE, Li Z, Chytil A, Geng Y, Gray JW, Moses HL, Yang L (2010). Gr-1+CD11b+ myeloid cells tip the balance of immune protection to tumor promotion in the premetastatic lung. Cancer Res.

[19] Khandoga A, Biberthaler P, Enders G, Krombach F (2004). 5-Aminoisoquinolinone, a novel inhibitor of poly (adenosine disphosphate-ribose) polymerase, reduces microvascular liver injury but not mortality rate after hepatic ischemia-reperfusion. Crit Care Med.

[20] Hatachi G, Tsuchiya T, Miyazaki T, Matsumoto K, Yamasaki N, Okita N, Nanashima A, Higami Y, Nagayasu T (2014). The poly (adenosine diphosphate-ribose) polymerase inhibitor PJ34 reduces pulmonary ischemia-reperfusion injury in rats. Transplantation.

[21] Liu Y, Zhang Y, Zhao Y, Gao D, Xing J, Liu H (2016). High PARP-1 expression is associated with tumor invasion and poor prognosis in gastric cancer. Oncology Lett.

[22] Nicoud IB, Jones CM, Pierce JM, Earl TM, Matrisian LM, Chari RS, Gorden DL (2007). Warm hepatic ischemia-reperfusion promotes growth of colorectal carcinoma micrometastases in mouse liver via matrix metalloproteinase-9 induction. Cancer Res.

[23] Psaila B, Lyden D (2009). The metastatic niche: adapting the foreign soil. Nat Rev Cancer.

[24] Lu L, Zhou H, Ni M, Wang X, Busuttil R, Kupiec-Weglinski J, Zhai Y (2016). Innate immune regulations and liver ischemia-reperfusion injury. Transplantation.

[25] Liu Y, Cao X (2016). Characteristics and significance of the pre-metastatic niche. Cancer cell.

[26] Inoue Y, Shirasuna K, Kimura H, Usui F, Kawashima A, Karasawa T, Tago K, Dezaki K, Nishimura S, Sagara J, Noda T, Iwakura Y, Tsutsui H (2014). NLRP3 regulates neutrophil functions and contributes to hepatic ischemia-reperfusion injury independently of inflammasomes. J Immunol.

[27] Christoffersson G, Vagesjo E, Vandooren J, Liden M, Massena S, Reinert RB, Brissova M, Powers AC, Opdenakker G, Phillipson M (2012). VEGF-A recruits a proangiogenic MMP-9-delivering neutrophil subset that induces angiogenesis in transplanted hypoxic tissue. Blood.

[28] Frangogiannis NG (2012). Regulation of the inflammatory response in cardiac repair. Circ Res.

[29] Kaplan RN, Rafii S, Lyden D (2006). Preparing the “soil”: the premetastatic niche. Cancer Res.

[30] Schriewer JM, Peek CB, Bass J, Schumacker PT (2013). ROS-mediated PARP activity undermines mitochondrial function after permeability transition pore opening during myocardial ischemia-reperfusion. J Am Heart Assoc.

[31] Greco R, Tassorelli C, Mangione AS, Levandis G, Certo M, Nappi G, Bagetta G, Blandini F, Amantea D (2014). Neuroprotection by the PARP inhibitor PJ34 modulates cerebral and circulating RAGE levels in rats exposed to focal brain ischemia. Eur J Pharmacol.

[32] Zhao H, Ning J, Lemaire A, Koumpa FS, Sun JJ, Fung A, Gu J, Yi B, Lu K, Ma D (2015). Necroptosis and parthanatos are involved in remote lung injury after receiving ischemic renal allografts in rats. Kidney Int.

[33] Chatterjee PK, Chatterjee BE, Pedersen H, Sivarajah A, McDonald MC, Mota-Filipe H, Brown PA, Stewart KN, Cuzzocrea S, Threadgill MD, Thiemermann C (2004). 5-Aminoisoquinolinone reduces renal injury and dysfunction caused by experimental ischemia/reperfusion. Kidney Int.

[34] Ledermann J, Harter P, Gourley C, Friedlander M, Vergote I, Rustin G, Scott C, Meier W, Shapira-Frommer R, Safra T, Matei D, Macpherson E, Watkins C, Carmichael J, Matulonis U (2012). Olaparib maintenance therapy in platinum-sensitive relapsed ovarian cancer. N Engl J Med.

[35] Rugo HS, Olopade OI, DeMichele A, Yau C, van 't Veer LJ, Buxton MB, Hogarth M, Hylton NM, Paoloni M, Perlmutter J, Symmans WF, Yee D, Chien AJ (2016). Adaptive randomization of veliparib-carboplatin treatment in breast cancer. N Engl J Med.

[36] Atretkhany KN, Drutskaya MS, Nedospasov SA, Grivennikov SI, Kuprash DV (2016). Chemokines, cytokines and exosomes help tumors to shape inflammatory microenvironment. Pharmacol Ther.

[37] Kowanetz M, Wu X, Lee J, Tan M, Hagenbeek T, Qu X, Yu L, Ross J, Korsisaari N, Cao T, Bou-Reslan H, Kallop D, Weimer R (2010). Granulocyte-colony stimulating factor promotes lung metastasis through mobilization of Ly6G+Ly6C+ granulocytes. Proc Natl Acad Sci U S A.

[38] Nastos C, Kalimeris K, Papoutsidakis N, Tasoulis MK, Lykoudis PM, Theodoraki K, Nastou D, Smyrniotis V, Arkadopoulos N (2014). Global consequences of liver ischemia/reperfusion injury. Oxid Med Cell Longev.

[39] Jaeschke H, Smith CW (1997). Mechanisms of neutrophil-induced parenchymal cell injury. J Leukoc Biol.

[40] Zhang P, Nakatsukasa H, Tu E, Kasagi S, Cui K, Ishikawa M, Konkel JE, Maruyama T, Wei G, Abbatiello B, Wang ZQ, Zhao K, Chen W (2013). PARP-1 regulates expression of TGF-beta receptors in T cells. Blood.

[41] Riley JP, Kulkarni A, Mehrotra P, Koh B, Perumal NB, Kaplan MH, Goenka S (2013). PARP-14 binds specific DNA sequences to promote Th2 cell gene expression. PLoS One.

[42] Ghonim MA, Pyakurel K, Ibba SV, Al-Khami AA, Wang J, Rodriguez P, Rady HF, El-Bahrawy AH, Lammi MR, Mansy MS, Al-Ghareeb K, Ramsay A, Ochoa A (2015). PARP inhibition by olaparib or gene knockout blocks asthma-like manifestation in mice by modulating CD4(+) T cell function. J Transl Med.

[43] Li MR, Chen GH, Cai CJ, Wang GY, Zhao H (2011). High hepatitis B virus DNA level in serum before liver transplantation increases the risk of hepatocellular carcinoma recurrence. Digestion.

[44] Li CX, Ling CC, Shao Y, Xu A, Li XC, Ng KT, Liu XB, Ma YY, Qi X, Liu H, Liu J, Yeung OW, Yang XX (2016). CXCL10/CXCR3 signaling mobilized-regulatory T cells promote liver tumor recurrence after transplantation. J Hepatol.

[45] Shi JH, Huitfeldt HS, Suo ZH, Line PD (2011). Growth of hepatocellular carcinoma in the regenerating liver. Liver Transpl.

[46] Bamboat ZM, Ocuin LM, Balachandran VP, Obaid H, Plitas G, DeMatteo RP (2010). Conventional DCs reduce liver ischemia/reperfusion injury in mice via IL-10 secretion. J Clin Invest.

[47] Yang L, DeBusk LM, Fukuda K, Fingleton B, Green-Jarvis B, Shyr Y, Matrisian LM, Carbone DP, Lin PC (2004). Expansion of myeloid immune suppressor Gr+CD11b+ cells in tumor-bearing host directly promotes tumor angiogenesis. Cancer Cell.

[48] Wang S, Shi XL, Feng M, Wang X, Zhang ZH, Zhao X, Han B, Ma HC, Dai B, Ding YT (2016). Puerarin protects against CCl4-induced liver fibrosis in mice: possible role of PARP-1 inhibition. Int Immunopharmacol.

[49] Schrotzlmair F, Kopitz C, Halbgewachs B, Lu F, Algul H, Brunner N, Gansbacher B, Kruger A (2010). Tissue inhibitor of metalloproteinases-1-induced scattered liver metastasis is mediated by host-derived urokinase-type plasminogen activator. J Cell Mol Med.

[50] Iyer RP, Patterson NL, Zouein FA, Ma Y, Dive V, de Castro Bras LE, Lindsey ML (2015). Early matrix metalloproteinase-12 inhibition worsens post-myocardial infarction cardiac dysfunction by delaying inflammation resolution. Int J cardiol.

[51] Arlt M, Kopitz C, Pennington C, Watson KL, Krell HW, Bode W, Gansbacher B, Khokha R, Edwards DR, Kruger A (2002). Increase in gelatinase-specificity of matrix metalloproteinase inhibitors correlates with antimetastatic efficacy in a T-cell lymphoma model. Cancer Res.

[52] Schelter F, Gerg M, Halbgewachs B, Schaten S, Gorlach A, Schrotzlmair F, Kruger A (2010). Identification of a survival-independent metastasis-enhancing role of hypoxia-inducible factor-1alpha with a hypoxia-tolerant tumor cell line. J Biol Chem.

[53] Shim JH, Park JY, Lee MG, Kang HH, Lee TR, Shin DW (2013). Human dermal stem/progenitor cell-derived conditioned medium ameliorates ultraviolet a-induced damage of normal human dermal fibroblasts. PLoS One.

[54] Bai P, Canto C, Oudart H, Brunyanszki A, Cen Y, Thomas C, Yamamoto H, Huber A, Kiss B, Houtkooper RH, Schoonjans K, Schreiber V, Sauve AA (2011). PARP-1 inhibition increases mitochondrial metabolism through SIRT1 activation. Cell Metabol.

[55] Lee CH, Syu SH, Liu KJ, Chu PY, Yang WC, Lin P, Shieh WY (2015). Interleukin-1 beta transactivates epidermal growth factor receptor via the CXCL1-CXCR2 axis in oral cancer. Oncotarget.

