# Supplementary material for: PARP-1 promotes tumor recurrence after warm ischemic liver graft transplantation via neutrophil recruitment and polarization
Source: Oncotarget. 2017 Oct 4;8(51):88918–33. doi: 10.18632/oncotarget.21493 (PMC5687657; doi:10.18632/oncotarget.21493)
Supplement: Supplementary file 1 [file oncotarget-08-88918-s001.pdf]

PARP-1 promotes tumor recurrence after warm ischemic liver graft transplantation via neutrophil recruitment and polarization

SUPPLEMENTARY MATERIALS

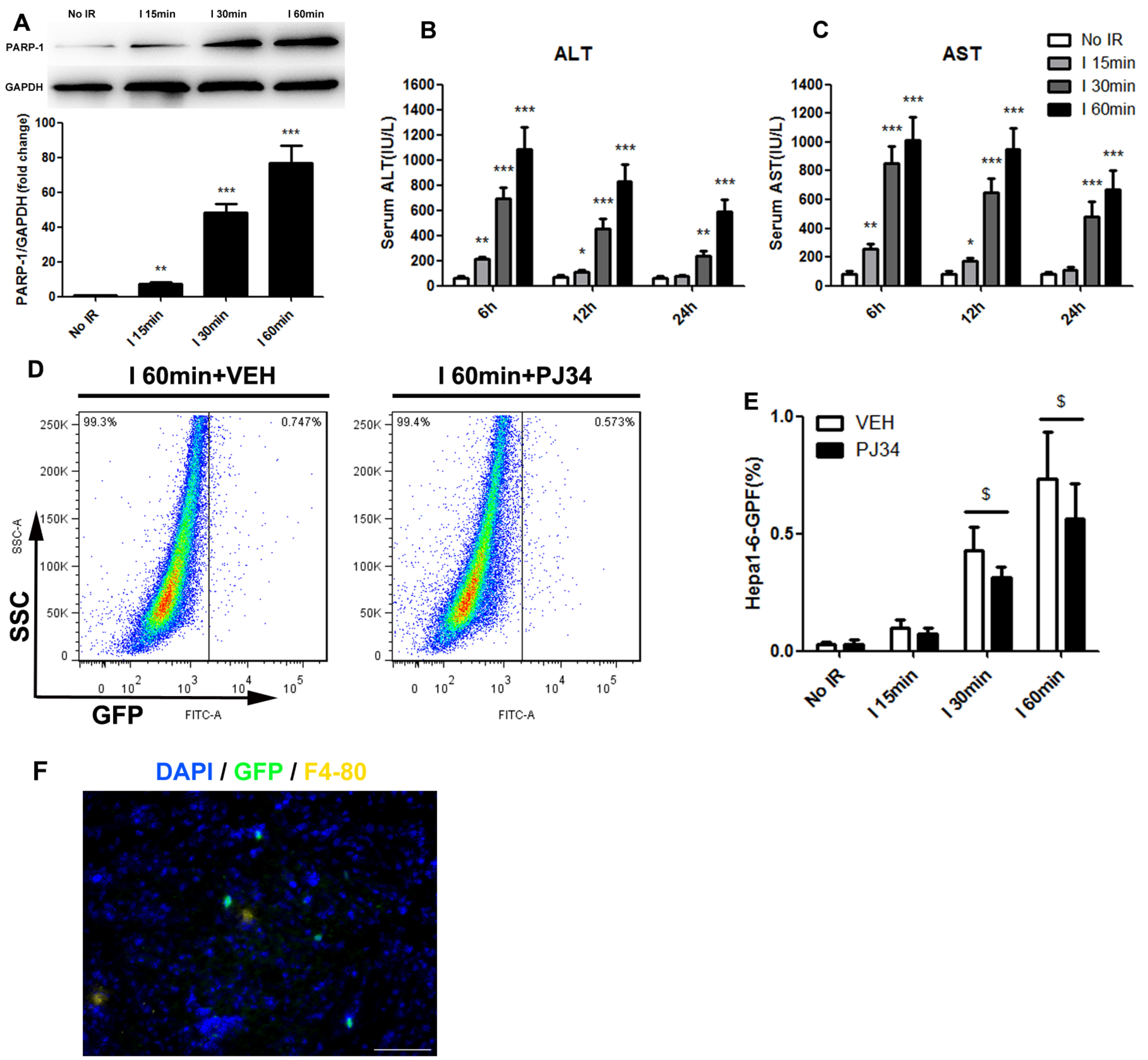

Supplementary Figure 1: (A) Up-regulation of PARP-1 expression induced by hepatic warm IR injury was detected by western blot. **(B, C)** The levels of ALT and AST were elevated and reached peak levels at 6h reperfusion (n=3-5 per group). **(D, E)** Syngeneic mouse HCC cell Hepa1-6-GPF implantation rates in single-cell suspension from ischemic liver lobes were detected by flow cytometry after 12h reperfusion (n=6-8 per group). **(F)** CBRH-7919-GFP was free cell in the liver instead of being phagocyted by macrophages cells (Ischemic time=30min, reperfusion time=12h, n=5, bar=100μm). mean ± SEM are shown. *p<0.05, **p<0.01, ***p<0.001 compared to the No IR group. $^{\$}$p<0.05, $^{\$\$}$p<0.01, $^{\$\$\$}$p<0.001. Data were analyzed by either Student's t-test or by ANOVA as appropriate followed by Bonferroni post-tests.

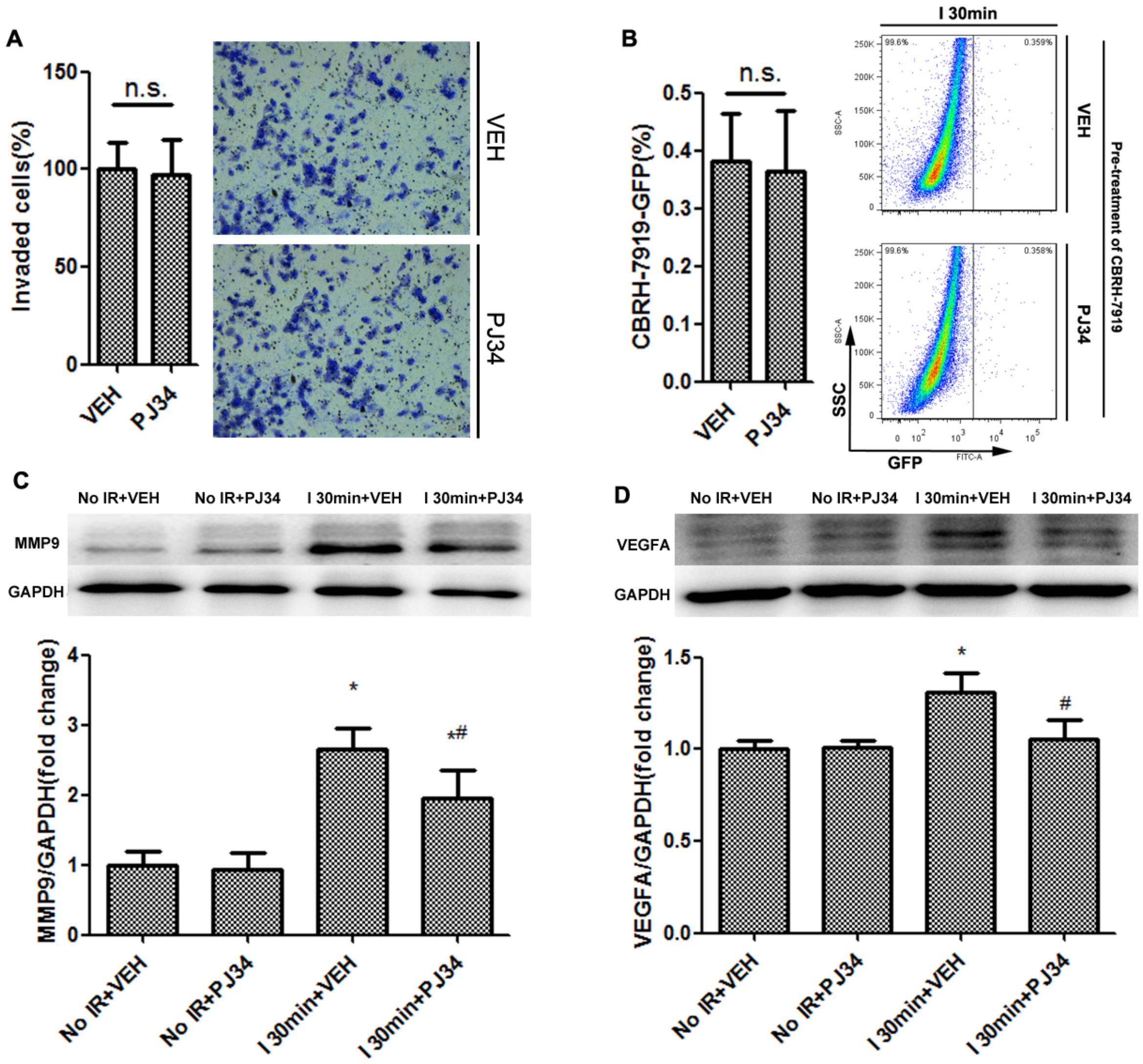

Supplementary Figure 2: Pretreatment with PJ34 for CBRH-7919-GFP had no effect on *in vitro* transwell invasion **(A)** or *in vivo* hepatic invading rates **(B).** Expression of MMP9 **(C)** and VEGFA **(D)** in liver after IR injury in VEH- or PJ34-treated groups was determined by western blot. mean ± SEM are shown. *$p<0.05$ compared to the No IR group. $^{\#}p<0.05$ compared to the I 30min+VEH group. n.s., not significant; Data were analyzed by either Student's t-test or by ANOVA as appropriate followed by Bonferroni post-tests.

A

	A	B	C	D	E	F	G	H
1<br>2	POS	POS	NEG	NEG	6Ckine (CCL21)	BLC (CXCL13)	CTACK (CCL27)	CXCL16
3<br>4	Eotaxin-1 (CCL11)	Eotaxin-2 (CCL24)	Fractalkine (CX3CL1)	I-TAC (CXCL11)	KC (CXCL1)	LIX	MCP-1 (CCL2)	MCP-5
5<br>6	MDC (CCL22)	MIG (CXCL9)	MIP-1 alpha (CCL3)	MIP-1 gamma	MIP-2	MIP-3 beta (CCL19)	MIP-3 alpha (CCL20)	PF-4 (CXCL4)
7<br>8	RANTES (CCL5)	SDF-1 (CXCL12)	TARC (CCL17)	I-309 (TCA-3/CCL1)	TECK (CCL25)	BLANK	BLANK	POS

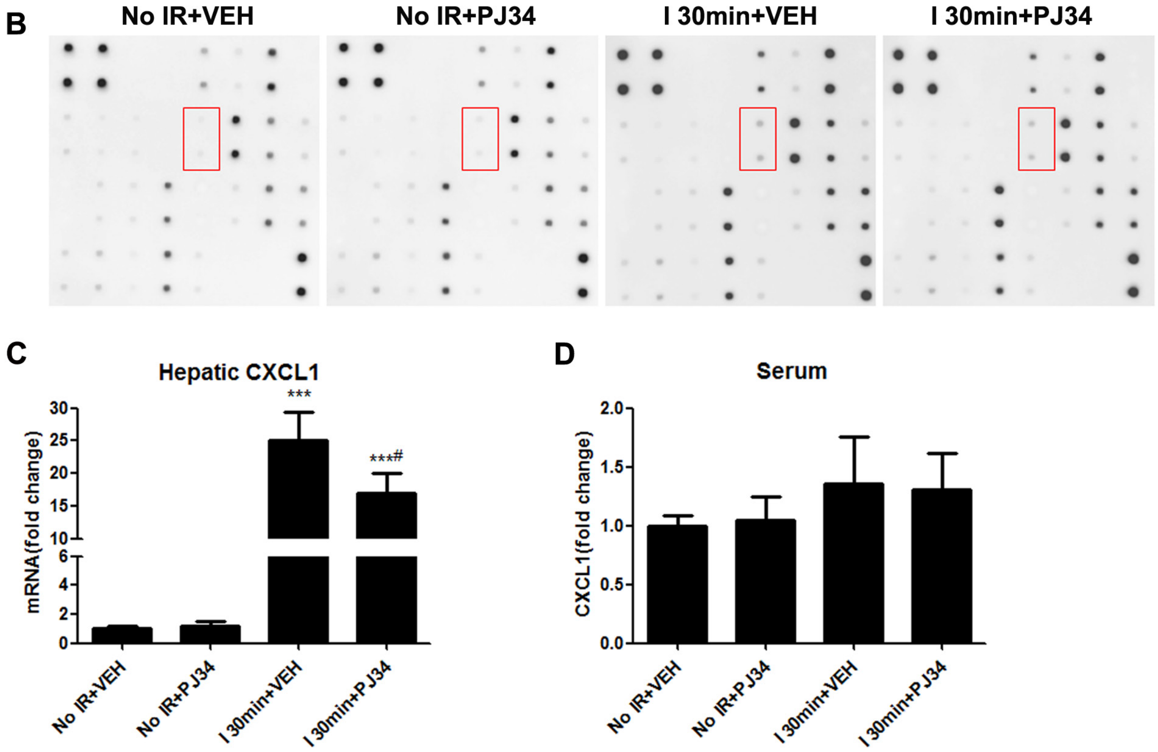

Supplementary Figure 3: (A) Map of antibodies against chemokines on the RayBiotech Mouse Chemokine Antibody Array (AAM-CHE-1). **(B)** Tissue lysates from ischemic liver lobes were applied to the array. Cytokine of significant difference was labeled with red frames. The levels of CXCL1 in liver tissues **(C)** and serum **(D)** were detected. mean ± SEM are shown. *p<0.05, **p<0.01, ***p<0.001 compared to the No IR group. $^{\#}$p<0.05 compared to the I 30min+VEH group; Data were analyzed by ANOVA as appropriate followed by Bonferroni post-tests.

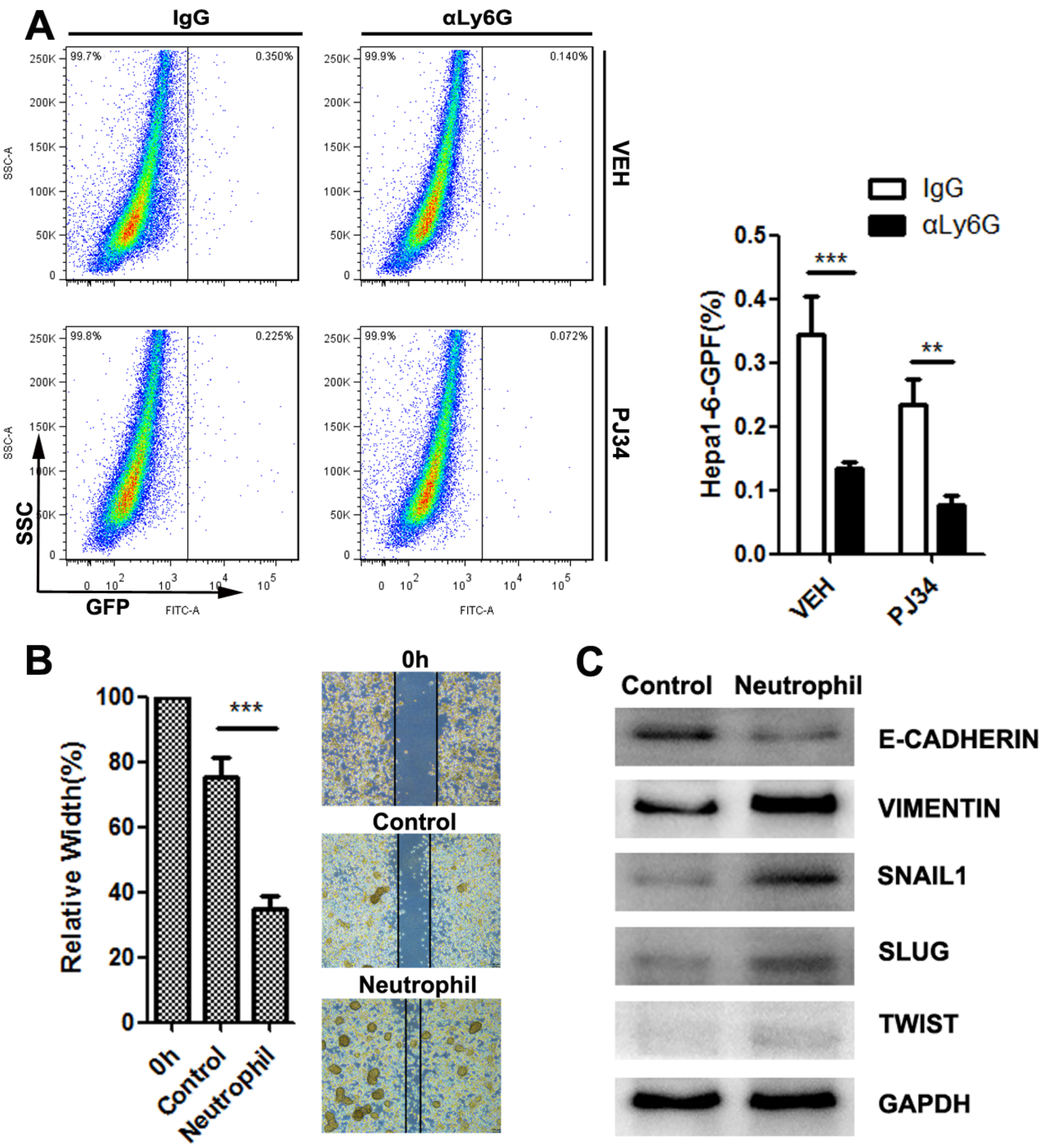

Supplementary Figure 4: (A) Hepatic recurrence burden with Hepa1-6-GFP after anti-Ly6G antibody injection was analyzed by flow cytometry in I 30min groups with or without PARP-1 inhibition (n=6-8 per group). **(B)** In the wound-healing assay, CBRH-7919 cells were kept in FBS-free medium and co-cultured with freshly isolated neutrophils after being starved for 24h. Imaged were taken at 24 after wounding. **(C)** Alterations of EMT-associated protein expression in CBRH-7919 co-cultured with neutrophils were determined by western blot. mean ± SEM are shown. ***$p<0.001$; Student's t-test.

Supplementary Table 1: List of primers used in this study

Primers	Forward	Reverse
TGFβ1	GAAGTGGATCCACGAGCCCAAG	GCTGCACTTGCAGGAGCGCAC
Fibronectin1	GCTTTGGCAGTGGTCATTTCAG	ATTCCCGAGGCATGTGCAG
VEGF	GGAGACTCTTCGAGGAGCACTT	GGCGATTTAGCAGCAGATATAAGAA
S100A8	TGCGATGGTGATAAAGTGG	GGCCAGAAGCTCTGCTACTC
TNFα	AGGGTCTGGGCCATAGAACT	CCACCACGCTCTTCTGTCTAC
IL6	ACCAGAGGAAATTTTCAATAGGC	TGTGCACTTGCAGAAAACA
IL1β	GGTCAAAGGTTTGGAAGCAG	TGTGAAATGCCACCTTTTGA
IL10	CCAAGCCTTATCGGAAATGA	TTTTCACAGGGGAGAAATCG
CXCL1	TCTCCGTTACTTGGGGACAC	CCACACTCAAGAATGGTCGC
GAPDH	TGTGTCCGTCGTGGATCTGA	TTGCTGTTGAAGTCGCAGGAG
